# Hybrid Toffoli gate on photons and quantum spins

**DOI:** 10.1038/srep16716

**Published:** 2015-11-16

**Authors:** Ming-Xing Luo, Song-Ya Ma, Xiu-Bo Chen, Xiaojun Wang

**Affiliations:** 1 Information Security and National Computing Grid Laboratory, Southwest Jiaotong University, Chengdu 610031, China; 2 School of Mathematics and Statistics, Henan University, Kaifeng 475004, China; 3 State Key Laboratory of Networking and Switching Technology, Beijing University of Posts and Telecommunications, Beijing 100876, China; 4 School of Electronic Engineering, Dublin City University, Dublin 9, Ireland

## Abstract

Quantum computation offers potential advantages in solving a number of interesting and difficult problems. Several controlled logic gates, the elemental building blocks of quantum computer, have been realized with various physical systems. A general technique was recently proposed that significantly reduces the realization complexity of multiple-control logic gates by harnessing multi-level information carriers. We present implementations of a key quantum circuit: the three-qubit Toffoli gate. By exploring the optical selection rules of one-sided optical microcavities, a Toffoli gate may be realized on all combinations of photon and quantum spins in the QD-cavity. The three general controlled-NOT gates are involved using an auxiliary photon with two degrees of freedom. Our results show that photons and quantum spins may be used alternatively in quantum information processing.

Quantum computing is an active area of research because of its ability to efficiently solve difficult problems without efficient classical algorithms^1^,^2^,^3^,^4^. The quantum computer, the elementary quantum element in quantum applications, is still difficult to realize with the methods of modern science. Based on the qubit system in two-dimensional Hilbert space, most quantum algorithms^1^,^2^,^3^,^4^ require a large number of qubits to encode information^5^,^6^,^7^. These quantum algorithms may be realized by special quantum circuits consisting of basic gates corresponding to unitary matrices. In other words, the design of quantum algorithms is equivalent to the decomposition of a unitary matrix into a product of matrices chosen from a basic set^8^,^9^. From classical matrix decomposition, such as cosine-sine decomposition^9^, multiple controlling logic gates have been fundamental to the multiple-qubit evolution. Finding efficient ways to synthesize these controlling logic gates may allow large-scale quantum computing tasks to be performed on a shorter time-scale.

Because classical computing is designed around irreversible gates, it is impossible to directly translate this expertise into the quantum world. The Gottesman-Knill Theorem says that Clifford gates (CNOT, Hadamard, *S*) can be classically simulated efficiently, so they are probably not sufficiently universal for quantum computation. These gates, together with other one-qubit gates, not generated by the gates in the Clifford group, form a universal set of gates for quantum computation^10^. Based on classical reversible logic^11^, the Toffoli gate^8^,^9^ has played a central role in this field; it is a controlled controlled-NOT acting on three bits. The Toffoli gate is also of interest in other quantum applications, for example, as a building block in phase estimation^12^, error correction^13^, and fault tolerant quantum circuits^14^. Much progress has been made, and various physical architectures have been used, including NMR systems^13^, ion traps^15^,^16^, linear optics^17^, superconductors^18^ and atoms^19^,^20^. These experiments may create opportunities to investigate efficient quantum circuits for synthesizing quantum operations.

Qubit-based quantum applications require a two-level structure on atom, ion or photon systems that naturally have many accessible degrees of freedom (DOFs). These DOFs may be regarded as high-dimensional systems. In fact, high-dimensional systems may provide different quantum correlations and may be useful in quantum information processing^21^,^22^,^23^,^24^,^25^,^26^,^27^,^28^,^29^. High-dimensional systems are flexible in terms of improvements to the channel capacity^21^,^22^ and communication security^24^,^25^. Moreover, they also provide an alternate way of scaling quantum computation. By extending a proposal^29^, Lanyon *et al.*^30^ recently demonstrated a general technique that harnesses multi-level information carriers to significantly reduce the realization complexity of multiple-control logic gates. By making use of a multiple-level target system, they showed that the Toffoli gate and general two-qubit controlled-unitary gates may be realized with linear optics. Regrettably, their multiple-level target system is unscalable for large-scale applications such as Shor’s algorithm. This flaw is then addressed by using multiple-level auxiliary states^31^, which may result in a high-dimensional quantum Fourier transformation.

Motivated by their scheme^23^,^29^,^30^,^31^, in this paper, we propose modified proposals of the Toffoli gate by using auxiliary photons with two DOFs as an auxiliary four-dimensional quantum state. Previous results have shown that two DOFs of photons may be used to fuse hybrid quantum information^32^, reduce quantum resources^33^,^34^,^35^, and construct a universal ququart quantum computer^36^. Our application using two DOFs of photons is for the scalability of qubit-based quantum computations^23^,^30^ and to avoid high-dimensional quantum Fourier transformations^31^. Moreover, from the strong field provided by a Fabry-Perot-type cavity, cavity QED may have a very strong effect even at the single photon level. This effect is very useful for large-scale quantum computation. In fact, by exploring the giant optical circular birefringence induced by quantum-dot spins in one-sided optical microcavities^32^,^33^,^37^,^38^,^39^,^40^,^41^,^42^,^43^,^44^,^45^, a spin may be interacted with a linearly circularly polarized photon. Based on the cavity QED, the Toffoli gate can be deterministically implemented on all combinations of photons and spins using an auxiliary photon with the polarization DOF and the spatial mode DOF. Our schemes extend previous schemes^13^,^14^,^15^,^16^,^17^,^19^,^20^,^34^,^35^ with six CNOT gates, recent proposals^29^,^30^,^31^ with three CNOT gates and the multiple-level logic state. All of our input quantum systems are qubits. The multiple-dimensional system, i.e., one photon with two DOFs, is used as an auxiliary system to carry the control information^30^. With these constructions, the multiple DOFs will not cause confusion in quantum information processing due to different dimensions of encoded quantum systems^31^. The disentangling operations only involve single photon operations and detectors^31^. Furthermore, our Toffoli gate may be realized on all combinations of photons and quantum spins. Thus they may be very useful for hybrid quantum information processing from recent experiments^44^,^45^,^46^,^47^,^48^,^49^,^50^,^51^,^52^,^53^,^54^.

## Results

The Toffoli gate is an important three-qubit entangling gate in quantum logic gates^11^,^12^,^13^. It will flip the target qubit conditional on the two control qubits. Combined with the one-qubit Hadamard, the Toffoli gate offers a simple universal quantum gate set in comparison to the CNOT gate and one-qubit rotations^10^,^55^. Generally, a Toffoli requires at least five two-qubit gates or six CNOT gates^11^,^54^. If an additional logic state is permitted for the target, a reduced decomposition requires only three two-qubit gates^29^,^30^,^31^. The enhanced decomposition is achieved by harnessing a third level of the target information carrier, i.e., a qutrit with logical states 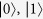 and 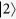. Motivated by this idea^29^,^30^,^31^, two DOFs of one photon as a multiple-dimensional system will be used as the control information carrier but not the target information carrier. Four logic states 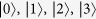 are encoded with 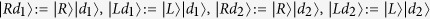, respectively. 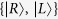 and 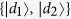 denote bases of the polarization DOF and spatial mode DOF of one photon respectively, where 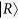 and 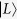 denote right and left circularly polarizing photons, respectively, and *d*_*i*_ denotes the spatial modes of one photon. In the following, we also denote 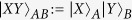 with 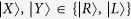 or 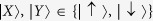 for convenience. By exploring the interaction of quantum-dot spins and a circularly polarized photon^32^,^33^,^37^,^38^,^39^,^40^,^41^,^42^,^43^,^44^,^45^, a Toffoli gate may be realized on the spins and photons regardless of the type of control and target qubits, using three general CNOT gates. These hybrid CNOT gates are typical controlling flip operations on the different DOFs of one photon or different types of quantum systems. These schemes show hybrid implementations of the Toffoli gate with photons and quantum spins using a reduced number of controlling qubit gates.

### QD-cavity system

**Figure 1:**
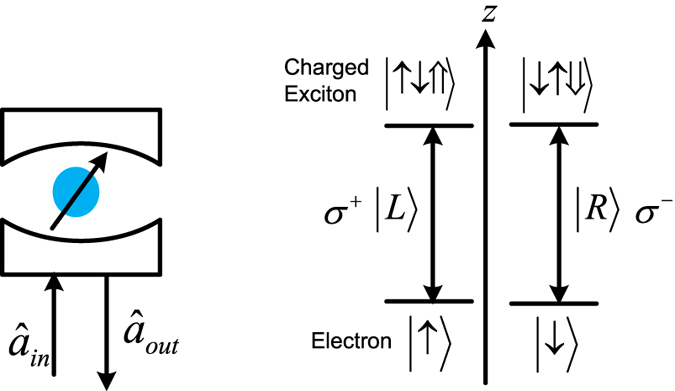
Schematic energy level and optical selection rules due to Pauli’s exclusion principle. 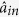
 and 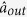 are the input and output field operators of the waveguide, respectively. 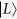 and 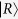 represent the left circularly and right circularly polarized photons, respectively.  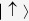 and 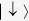 represent the spins of the excess electron.  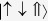 and 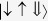 represent the negatively charged exciton *X*^−1^.

Consider a singly charged GaAs/InAs quantum dot (QD) inside a micropillar cavity^37^,^38^,^39^, which consists of a *λ*-cavity between two GaAs/Al(Ga)As distributed Bragg reflectors. The QD is located in the center of the cavity to achieve maximal light-matter coupling. If the QD is neutral, optical excitation generates a neutral exciton. If the QD is singly charged, i.e., a single excess electron is injected, optical excitation can create a negatively-charged exciton (*X*^−^), which consists of two electrons bound to one hole^37^,^38^,^39^. Due to Pauli’s exclusion principle, for the spin state 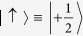, *X*^−^ in the state 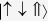 with the two electron spins antiparallel is created by resonantly absorbing a left circularly polarized photon 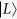, where the heavy-hole spin state 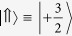; for the spin state 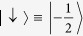, *X*^−^ in the state 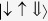 with the two electron spins antiparallel is created by resonantly absorbing a right circularly polarization photon 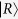, where heavy-hole spin state 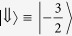, as shown in Fig. 1. In the limit of a weak incoming field^40^,^41^,^42^, the spin cavity system behaves like a beam splitter. Based on the transmission and reflection rules of the cavity for an incident circular polarization photon conditioned on the QD-spin state, the dynamics of the interaction between the photon and spin in a QD-microcavity coupled system is described as below^32^,^33^,^43^,^44^,^45^


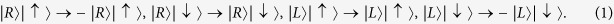


under ideal conditions. In the following, this ideal spin-cavity unit is used to realize the Toffoli gate on photons and quantum-dot spins for efficient quantum information processing. Then, the experimental spin-cavity unit will be discussed in the last section.

### Toffoli gate on a three-photon system

Consider three linearly circularly polarized photons *A*, *B* and *C* in the states


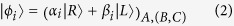


**Figure 2:**
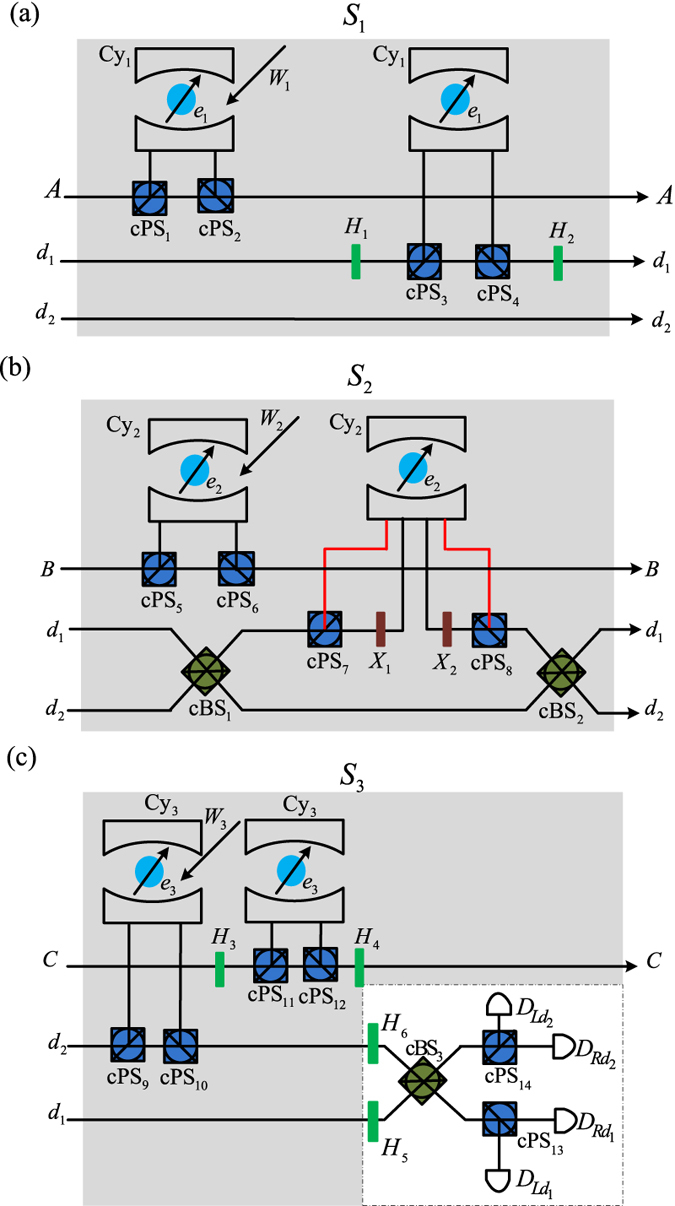
Toffoli gate on a three-photon system assisted by one photon with two DOFs. *d*_*i*_ denote spatial modes of the auxiliary photon *D*. *e*_*i*_ denote auxiliary electron spins in the state 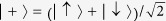. *H*_*i*_ denote half waveplates to perform the Hadamard transformation 
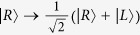
 and 
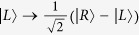
. *X*_*i*_ denote wave plates to perform the polarization flip transformation 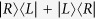. *Z*_*i*_ denote waveplates to perform the phase flip transformation 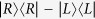. cPS_*i*_ represent circularly polarizing beamsplitters that transmit 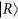 and reflect 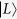. cBS_*i*_ represent 50%50 circularly polarizing beamsplitters to perform the Hadamard operation 
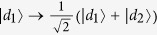
 and 
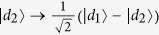
. Cy_*i*_ denote the QD-cavity charged the electron spin *e*_*i*_. If there are two input lines of one cavity, the photon represented with red lines passes through the cavity firstly, and then the photon represented with black lines passes through the cavity.

Our goal is to realize the Toffoli gate with the following form


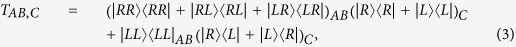


where the photons *A* and *B* are the controlling qubits while the photon *C* is the target photon. The detailed circuit is shown in Fig. 2. This construction is completed with three auxiliary quantum electron spins *e*_*i*_ in the state 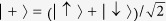 and an auxiliary photon *D* in the state 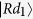. The Toffoli gate *T*_*AB*,*C*_ is completed with the following three controlled gates.

First, from the subcircuit *S*_1_ shown in Fig. 2(a), the photon *A* as an input pulse passes through the cPS_1_, cavity Cy_1_, cPS_2_, sequentially. Then *W*_1_ is performed on the spin *e*_1_. Now, the pulse *D* from the spatial mode *d*_1_ passes through the *H*_1_, cPS_3_, cavity Cy_1_, cPS_4_, *H*_2_, sequentially. After these operations, the joint system consisting of the photons *A* and *D*, and the spin *e*_1_ is changed from 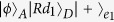 into 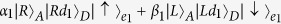; the detailed computations are shown in SI. This joint state may collapse into


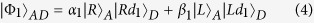


after the measurement of the electron spin *e*_1_ under the basis 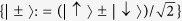, where a Pauli phase flip 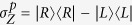 is performed on the photon *A* for the measurement outcome 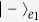. This circuit has realized the controlled-NOT gate 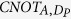 on the input photon *A* and the polarization DOF of the auxiliary photon *D*, which is different from previous CNOT gate on the same type of input system.

Second, from the subcircuit *S*_2_ shown in Fig. 2(b), the photon *B* passes through the cPS_5_, cavity Cy_2_, cPS_6_, sequentially. Then *W*_2_ is performed on the spin *e*_2_. Now, the photon *D* passes through the BS_1_, cPS_7_, *X*_1_, cavity Cy_2_, *X*_2_, cPS_8_, BS_2_, sequentially. After these operations, the joint system consisting of the photons *A*, *B* and *D*, and the spin *e*_2_ is changed from 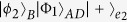 into 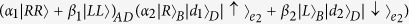; the detailed computations are shown in the SI. This state may collapse into


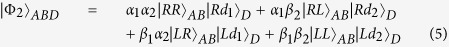


after the measurement of the electron spin *e*_2_ under the basis 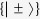, where a Pauli phase flip 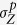 is performed on the photon *B* for the measurement outcome 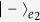. This circuit has realized the controlled-NOT gate 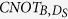 on the input photon *B* and the spatial mode DOF of the auxiliary photon *D*.

Third, from the subcircuit *S*_3_ shown in Fig. 2(c), the pulse *D* from the spatial mode *d*_2_ passes through the cPS_9_, cavity Cy_3_, cPS_10_, sequentially. Then *W*_3_ is performed on the spin *e*_3_ Now, the photon *C* passes through the *H*_3_, cPS_11_, cavity Cy_3_, cPS_12_, *H*_2_, sequentially. After these operations, the joint system consisting of the photons *A*, *B*, *C* and *D*, and the spin *e*_3_ is changed from 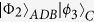 into


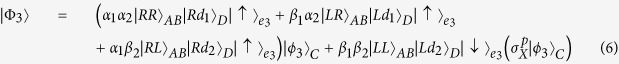


where a Pauli flip 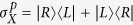. This state may collapse into


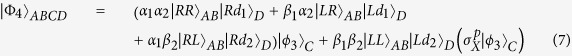


after the measurement of the spin *e*_3_ under the basis 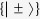, where a phase flip 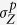 is performed on the photon *D* from the spatial mode *a*_2_ for the measurement outcome 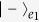. This circuit may be viewed as the controlled-NOT gate *CNOT*_*D*,*C*_ performed on the auxiliary photon *D* and the input photon *C* as follows


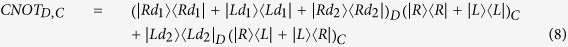


which is an essential three-qubit operation.

Finally, by performing the single qubit measurements on the photon *D* under the basis 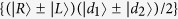. In the experiment, this measurement may be completed with the 50%50 circularly polarizing beamsplitter cBS_3_, two circularly polarizing beamsplitters cPS_13_ and cPS_14_, two half waveplates *H*_5_ and *H*_6_, and four single photon detectors 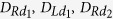 and 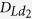. The recovery operations are shown in Table 1. The entanglement 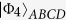 shown in equation (7) may collapse into


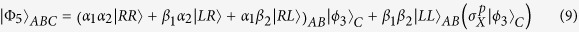


Thus, the Toffoli gate *T*_*AB*,*C*_ shown in equation (3) has been deterministically realized with three general controlled gates 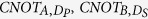 and *CNOT*_*D*,*C*_.

### Toffoli gate on a three-spin system

Consider three electron spins *e*_*i*_ in the states


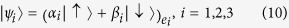


**Figure 3:**
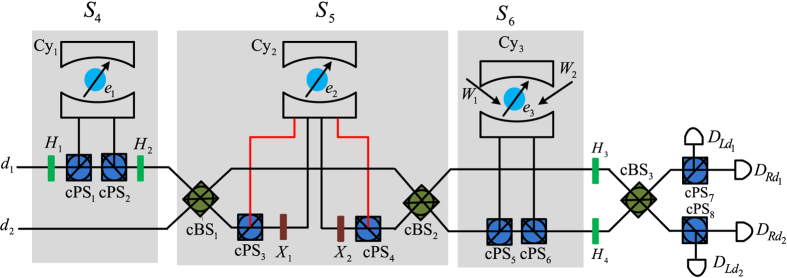
Toffoli gate on a three-spin system assisted by one photon with two DOFs. cPS_*i*_, cBS_*i*_, *X*_*i*_, *H*_*i*_ and *W*_*i*_ are the same as those defined in Fig. 2. *e*_*i*_ d*e*note input electron spins. *d*_*i*_ *d*enote spatial modes of an auxiliary photon *D* in the state 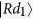.

This section is to realize the Toffoli gate


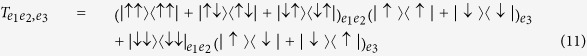


where the electron spins *e*_1_ and *e*_2_ are the controlling qubits, while the electron spin *e*_3_ is the target qubit. The detailed circuit is shown in Fig. 3 by using an auxiliary photon *D* in the state 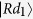. This Toffoli gate is realized with the following three controlled gates on electron spins.

First, the auxiliary photon *D* from the spatial mode *d*_1_ passes through the half waveplate *H*_1_ to *H*_2_ sequentially. The joint system consisting of the photon *D* and the electron spin *e*_1_ changes from 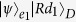 into


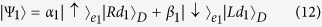


This subcircuit (denoted as *S*_4_) has realized the controlled-NOT gate 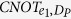 on the spin *e*_1_ and the polarization DOF of the auxiliary photon *D* under the joint basis 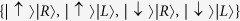.

Moreover, by letting the photon *D* pass the *cBS*_1_ to *cBS*_2_ sequentially, the joint system 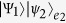 may be changed into


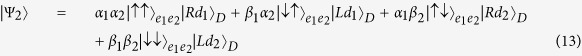


This subcircuit (denoted as *S*_5_) has realized the controlled-NOT gate 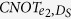 on the spin *e*_2_ and the spatial mode DOF of the photon *D* under the joint basis 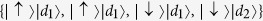.

Furthermore, let the photon *D* pass the *W*_1_ to *W*_2_ sequentially. The joint system 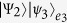 may be changed into


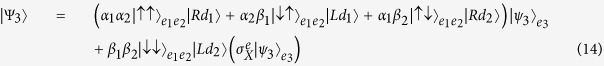


where the Pauli flip 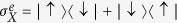. This subcircuit (denoted as *S*_6_) has realized the controlled-NOT gate 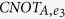 on the auxiliary photon *D* and the input spin *e*_3_ under the joint basis 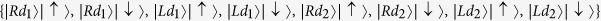.

Finally, the joint system 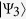 shown in the equation (14) may collapse into


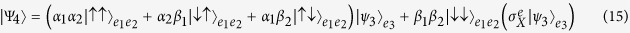


by measuring the auxiliary photon *D* under the basis 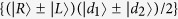. Similarly, this measurement may be implemented in the experiment with the 50%50 circularly polarizing beamsplitter cBS_3_, two circularly polarizing beamsplitters cPS_7_ and cPS_8_, two half waveplates *H*_3_ and *H*_4_, and four single photon detectors 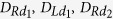 and 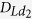. The recovery operations are shown in Table 2. Thus, the three-spin Toffoli gate 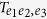 shown in the equation (13) has been deterministically realized with three control gates 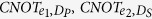 and 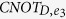.

### Toffoli gate on hybrid three-qubit systems

**Figure 4:**
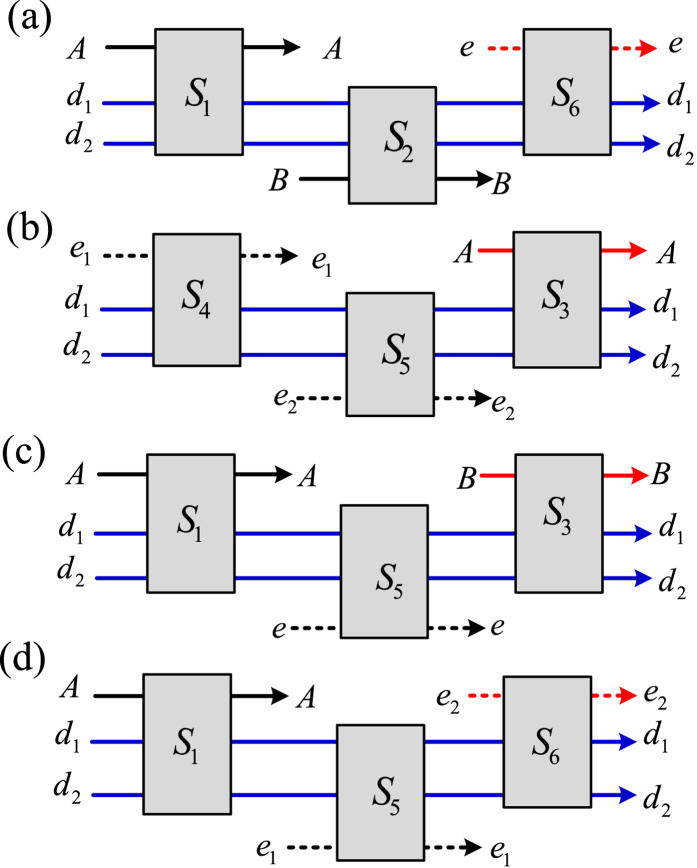
Toffoli gate on hybrid three-qubit systems assisted by one photon with two DOFs. (**a**) Two photons *A* and *B* jointly control an electron spin *e*. (**b**) Two electron spins *e*_1_ and *e*_2_ jointly control a photon *A*. (**c**) One photon *A* and one electron spin *e* jointly control a photon *B*. (**d**) One photon *A* and one electron spin *e*_1_ jointly control an electron spin *e*_2_. *S*_1_, *S*_2_ and *S*_3_ denote the subcircuits shown in Fig. 2(a–c), respectively. *S*_4_, *S*_5_ and *S*_6_ are shown in Fig. 3. The bule lines denote the controlling qubits while the red lines denote the target qubits. The black lines denote an auxiliary photon *D* in the state 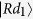. *M*_*D*_ denotes the measurement of the photon *D* shown in Fig. 2(c).

The present Toffoli gate on a three-photon system shown in the Fig. 2 and a three-spin system shown in Fig. 3 may be combined to realize Toffoli gate on hybrid three-qubit systems. Thus, the three input qubits may be an arbitrary combination of photons and quantum spins. Because of the symmetry of two control qubits, four different cases are to be considered, as shown in Fig. 4.

First, let two photons *A* and *B* jointly control an electron spin *e*; their initial states are 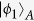, 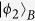 and 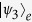, respectively. The detailed circuit is shown in Fig. 4(a). From the 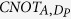 realized with the subcircuit *S*_1_, the joint system consisting of three qubits and an auxiliary photon *D* changes from 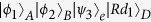 into


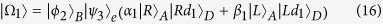


Moreover, from the CNOT gate realized with the subcircuit *S*_2_, 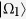 may change into


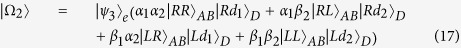


Furthermore, from the CNOT gate realized with the subciruit *S*_6_, the joint system 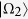 shown in the equation (17) changes into


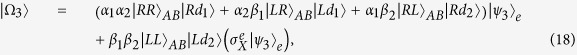


which may collapse into


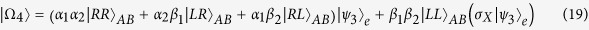


by performing the single qubit measurement *M*_*D*_ on the photon *D* under the basis 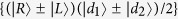. In the experiment, this measurement may be implemented in experiments with a 50%50 circularly polarizing beamsplitter, two circularly polarizing beamsplitters, two half waveplates, and four single photon detectors, as shown in Fig. 2(c). The recovery operations are similar to these shown in Table 1. Thus, a Toffoli gate has been realized on the two photons and one spin using three CNOT gates.

Second, consider two electron spins *e*_1_ and *e*_2_ in the states 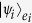 that jointly control one photon *A* in the state 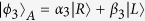. The detailed circuit is shown in Fig. 4(b). From the CNOT gates realized with the subcircuit *S*_4_ and *S*_5_ in Fig. 3, the joint system consisting of three input qubits and the auxiliary photon *D* changes from 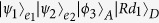 into


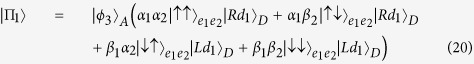


Moreover, from the CNOT realized with the subcircuit *S*_3_ in Fig. 2(c), the joint system 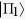 changes into


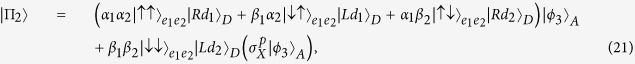


which may collapse into


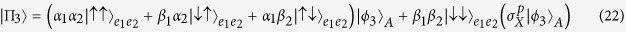


after performing the measurement *M*_*D*_ of the photon *D* under the basis 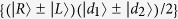. The recovery operations are shown in Table 2. Thus, a Toffoli gate has been realized on two electron spins and one photon.

Third, consider one photon *A* in the state 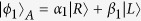, and one spin *e* in the state 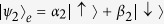 that jointly control one photon *B* in the state 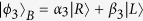. The detailed circuit is shown in Fig. 4(c). Similar to the subcircuits shown in Fig. 4(a,b), from the CNOT gates realized with the subcircuits *S*_1_ in Fig. 2(a), *S*_5_ in Fig. 3 and *S*_3_ in Fig. 2(c), the joint system of the three input qubits and the auxiliary photon *D* changes from 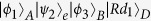 into


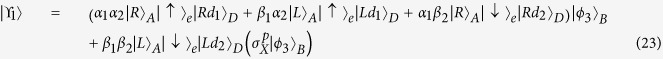


which may collapse into


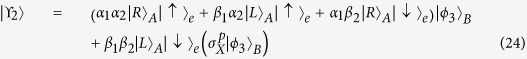


after the measurement *M*_*D*_ of the photon *D* under the basis 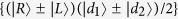. The recovery operations are shown in Table 2. The difference is that the Pauli phase flip 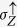 is performed on the controlling spin *e*. Thus, a Toffoli gate has been realized on two electron spins and one photon.

Finally, consider one photon *A* in the state 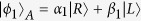 and one electron spin *e*_1_ in the state 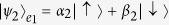 that jointly control the other electron spin *e*_2_ in the state 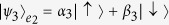. The detailed circuit is shown in Fig. 4(d). Similar to the subcircuit shown in Fig. 4(c), from the CNOT gates realized with the subcircuits *S*_1_ in Fig. 2(a), *S*_5_ in Fig. 3 and *S*_6_ in Fig. 3, the joint system consisting of three input qubits and the auxiliary photon *D* changes from 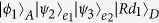 into


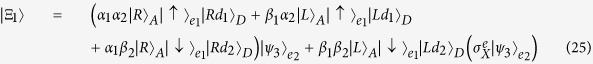


which may collapse into


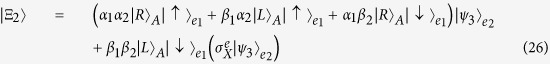


by performing the measurement *M*_*D*_ of the photon *D* under the basis 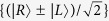 and 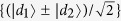 for the polarization DOF and spatial mode, respectively. The recovery operations are the same as those in Fig. 4(c). Thus, the spin qubit may be jointly controlled by one photon and one spin.

## Discussion

The optical selection rules of a QD-cavity system shown in equation (1) play core roles in the present Toffoli gates. In the resonance conditions Δ*ω*_*x*_ = Δ*ω*_*c*_ = 0, if one neglects the cavity side leakage *κ*_*s*_ ≈ 0, it easily follows that |*r*_0_| → 1 and |*r*| → 1 when the cooperativity parameter *g*^2^/(*κγ*) of cavity QED is large enough. Thus, our six Toffoli gates are deterministic and faithful. However, the side leakage from the cavity is unavoidable in the experiment^44^,^45^,^47^,^48^,^49^,^50^,^51^,^52^,^53^,^54^. In the following, consider two kinds of transition channels for the cavity photon. The first is the cavity decay due to transmission through the cavity mirror, whose rate is *κ*. Every other unwanted photon loss, such as cavity absorption and scattering, are characterized by the overall loss rate *κ*_*s*_. Taking into account the coupling through the cavity decay channel and neglecting the spatial dependence, the relation of the input field operator 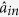 and output operator 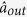 may be approximated with an experimental reflection coefficient^37^,^38^,^39^


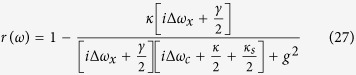


where Δ*ω*_*c*_ and Δ*ω*_*x*_ are the frequency detunings of the cavity mode and dipole transition, respectively, in relation to the input probe light (See Method). When the quantum dot is uncoupled from the cavity (*g* = 0), *r*(*ω*) is reduced to^37^,^38^,^39^


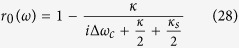


**Figure 5:**
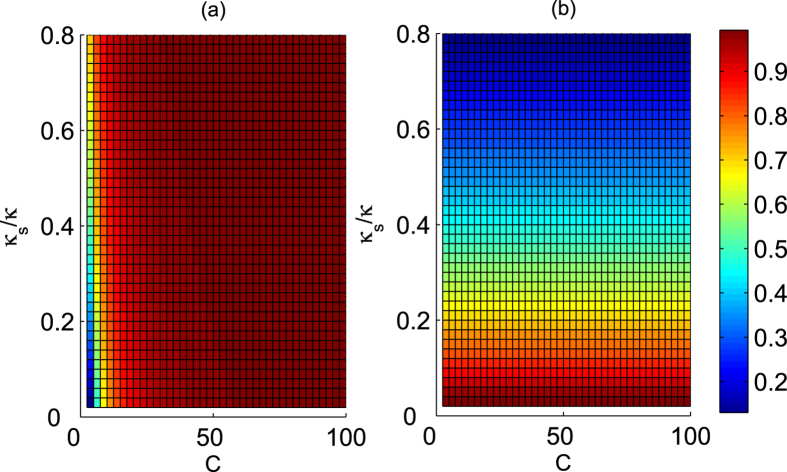
Reflection coefficients versus the cavity leakage ratio *κ*_*s*_/*κ* and the cooperativity *C* under resonant conditions. (**a**) Reflectance |*r*| and (**b**) reflectance |*r*_0_| under resonant conditions.

**Figure 6:**
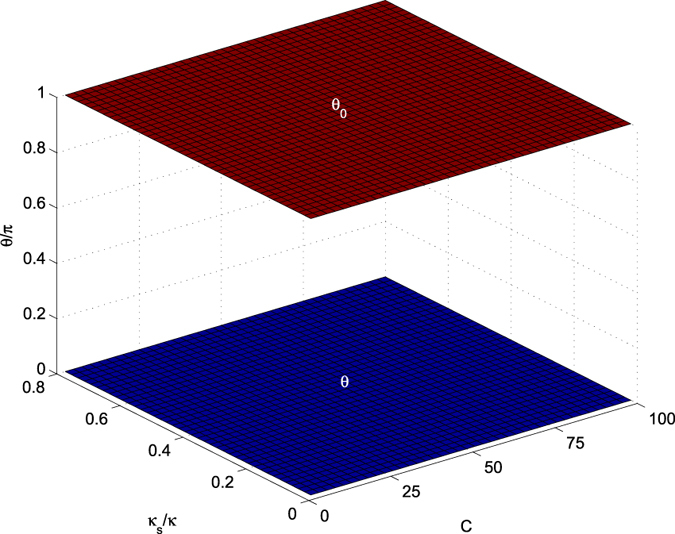
Phase shifts versus the cavity leakage ratio *κ*_*s*_/*κ* and the cooperativity *C* under resonant conditions. Here, the scale of the phase shift is *π*.

These complex coefficients indicate that the reflected light may experience a phase shift^32^,^33^,^34^,^35^,^36^,^44^,^45^,^46^. Under resonant conditions Δ*ω*_*c*_ = Δ*ω*_*x*_ = 0, the reflection coefficients |*r*| and |*r*_0_| are evaluated in Fig. 5, and the phase shifts *θ* and *θ*_0_ are evaluated in Fig. 6 inrelation to the decay ratios of cavity *κ*_*s*_/*κ* and the cooperativity parameter *C* = *g*^2^/(*κγ*) of cavity QED^56^,^57^, which is a geometric parameter that characterizes the absorptive, emissive, or dispersive coupling of an atom to the cavity mode. Based on Fig. 5, the reflection coefficients will satisfy |*r*| ≈ 1 and |*r*_0_| ≈ 1 when *C* ≫ 10 and *κ*_*s*_/*κ* → 0, and these additional conditions are not required for relative phase shifts *θ*_0_ = *π* and *θ* = *π* because *r* and *r*_0_ are real under the resonant conditions Δ*ω*_*c*_ = Δ*ω*_*x*_ = 0. Hence, the real reflection coefficients *r* and *r*_0_ will be considered under the resonant conditions.

In fact, the ideal optical selection rules shown in equation (1) are changed into


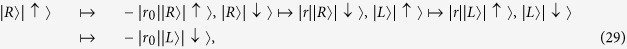


in the experiment. Based on these general optical selection rules, one can also complete the Toffoli gate from our schemes.

**Figure 7:**
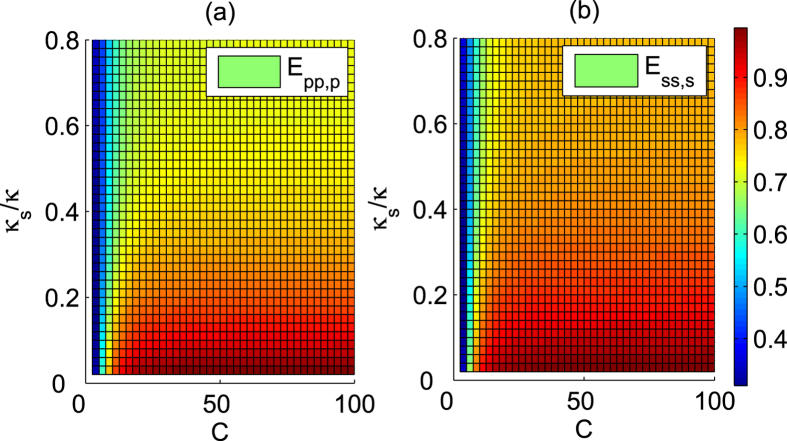
Efficiencies of Toffoli gate versus the cavity leakage ratio *κ*_*s*_/*κ* and the cooperativity *C*. (**a**) Efficiency *E*_*pp*,*p*_ of Toffoli gate on a three-photon system. (**b**) Efficiency *E*_*ss*,*s*_ of Toffoli gate on a three-spin system.

**Figure 8:**
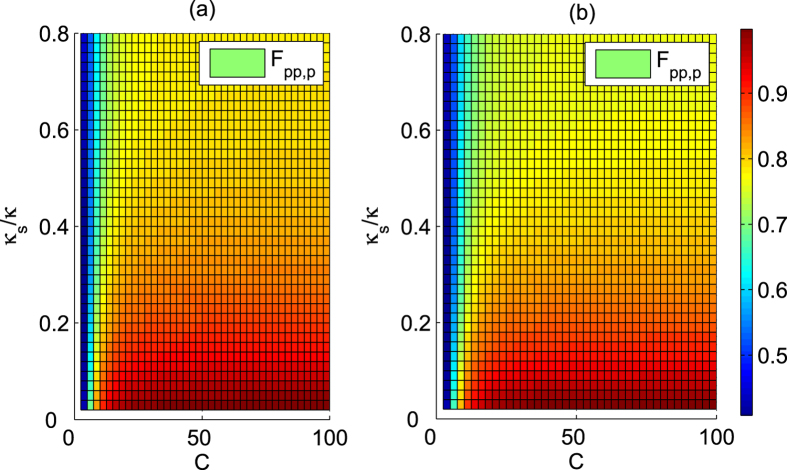
Average fidelities of Toffoli gate versus the cavity leakage ratio *κ*_*s*_/*κ* and the cooperativity *C*. (**a**) The average fidelity *F*_*pp*,*p*_ of Toffoli gate on a three-photon system. (**b**) The average fidelity *F*_*ss*,*s*_ of Toffoli gate on a three-spin system.

For our first Toffoli gate on the three photons shown in Fig. 2, three auxiliary electron spins *e*_1_, *e*_2_ and *e*_3_ in the state 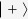 are used, and four photons *A*, *B*, *C*, and *D* are involved; the success of this protocol is heralded by the instance in which the detector 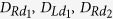 or 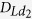 click. The efficiency of our Toffoli gate is defined by 
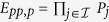
, where *P*_*j*_ is a successful reflection probability of the *j*-th photon from a micropillar cavity^37^,^50^,^54^,^57^, and 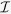 denotes the index set of photons involved in each scheme. Its efficiency is evaluated in Fig. 7(a). To detail the influence of the practical input-output process on the fidelity of the final joint system after this Toffoli gate, we take the case in which the detector 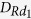 clicks as an example and obtain the average fidelity *F*_*pp*,*p*_, as evaluated in Fig. 8(a). Here, 
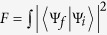
, where the integral is evaluated over all possible input states, 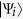 and 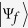 are the ideal final state and the experimental final state with side leakages, respectively. For our second Toffoli gate on three electron spins shown in Fig. 3, three electron spins *e*_1_, *e*_2_ and *e*_3_ are involved, and one photon *D* is used; its success is determined by the photon *D*, which is detected at the detector 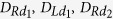 or 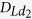 click. The practical efficiency *E*_*ss*,*s*_ is evaluated in Fig. 7(b) whereas the average fidelity *F*_*pp*,*p*_ is evaluated in Fig. 8(b) for the photon *D* detected at the detector 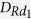 as an example. For the other four cases, one can obtain similar results.

Typically, the cavity side leakage may greatly affect the efficiency and fidelity of the Toffoli gate. As shown in the Figs 7 and 8, high efficiency and fidelity may be achieved even in the weakly coupling regime when 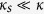. Otherwise, the strong coupling defined by *g* ≫ (*κ*, *γ*) is necessary^39^,^40^,^41^,^48^,^49^,^50^,^51^,^52^,^53^,^54^. The classical strong-coupling condition corresponds to the single-photon Rabi frequency 2*g* being larger than the geometric mean of the atomic and cavity line widths. In general, the system can be parameterized in terms of two dimensionless parameters, namely, the ratios *g*/*κ* and *g*/*γ* in the cavity QED description or, in the classical description, the cooperativity parameter *C* and the line width ratio *κ*/*γ*. The cavity QED strong-coupling condition 2*g* > (*κ*, *γ*) corresponds to a normal-mode splitting that is much larger than the line widths of the normal modes. The cooperativity parameter of cavity QED is shown to play a central role and is given a geometrical interpretation. The cooperativity has been realized up to 27^58^. Under this cooperativity, the efficiencies *E*_*PP*,*P*_ and *E*_*SS*,*S*_ are greater than 91.24% for *κ*_*s*_/*κ* ≈ 0.2^48^,^54^; the average fidelities *F*_*PP*,*P*_ and *F*_*SS*,*S*_ are greater than 93.47% for *κ*_*s*_/*κ* ≈ 0.2^48^,^54^. If one hopes to achieve a fault tolerance threshold of 7.5 × 10^−3^ on a two-dimensional lattice of qubits^59^, the cavity leakage ratio should be *κ*_*s*_/*κ* < 0.04 and the cooperativity should be *C* > 28 for a photonic Toffoli gate, whereas the cavity leakage ratio should be *κ*_*s*_/*κ* < 0.03 and the cooperativity should be *C* > 34 for a Toffoli gate on a three-spin system. When the fault tolerance threshold is reduced to 1 × 10^−3^ using controlled phase gates based on dipole-induced transparency^60^, the cavity leakage ratio should be reduced to 0.02, and the cooperativity should be improved to *C* > 38 for a photonic Toffoli gate, whereas the cavity leakage ratio should be reduced to 0.015 and the relative coupling strength should be improved to 4.2 for a Toffoli gate on a three-spin system. *κ*_*s*_/*κ* = 0.05 has been reported, which could be achieved by taking a pillar microcavity with the quality factor of *Q* = 165000 demonstrated in ref. 54 and decreasing the reflection of the top mirror to reduce the quality factor to *Q* = 9000, which is still in the strong-coupling regime^48^.

If the experimental electron spin decoherence and trion dephasing^41^,^42^ are considered, the real efficiency and fidelity are slightly decreased when the hole spin coherence time is longer than three orders of the cavity photon lifetime^44^,^50^,^51^. Moreover, by using the spin echo technique^57^,^61^ and the nanosecond spin resonance microwave pulse^47^ to protect the electron spin coherence, faithful Hadamard transformations may be implemented on the electron spin for our six Toffoli gates. The heavy-light hole mixing may be reduced by engineering the shape, size and type of the charged exciton^61^. The optical selection rule has been experimentally realized with the spin state of a single trapped atom and the polarization state^44^,^45^. To achieve weak excitation, some adiabatic conditions are used to ensure that the *X*^−^ stays in the ground state for the most time. With a first-order approximation, we can adiabatically eliminate 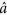 from the third subequation of equation (31) by substituting the steady-state solution to the first two subequations of equation (31). Under the adiabatic condition 
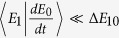
, the system may be unchanged between the ground state 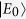 and excite state 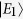 under the first-order approximation. Here, Δ*E*_10_ = *E*_1_ − *E*_0_. If the dephasing is considered for the atomic system, it may be modeled by introducing phenomenological decay terms or noise operators 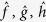 into three subequations of equation (31). Because the output modes are initially in a vacuum, the 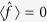. By substituting the steady-state solution to the third subequation of equation (31), the only difference is one noise operator 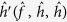 and the modified spontaneous emission rate of the dipole 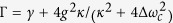
62. Of course, the present Toffoli schemes are also conditional on the perfect overlap of the cavity mode with the two spatially separated optical beams, the phase stability of the interferometer composed of the cBS, and the perfect time overlap of two beams passing through several interferometers.

In conclusion, we have investigated the possibility of hybrid quantum computation assisted by the quantum spins and photons with two DOFs. Six deterministic Toffoli gates are realized on the joint system of all combinations of the photon or the quantum spin systems. Compared with previous Toffoli gates^13^,^14^,^15^,^16^,^17^,^19^,^20^, our Toffoli gates may be realized with three general control-NOT gates, which are similar to the schemes in ref. 29, 30, 31. Unlike the multiple dimensional quantum target state of the photonic Toffoli gate^18^,^30^, all the input systems are qubit systems, whereas the additional multiple-dimension logic state is used as the auxiliary system. With the modification, one does not need to consider the different dimensional quantum systems to encode information in quantum applications. This method is similar to that in ref. 31. However, their disentangling operations are necessary and essential controlled operations or high-dimensional operations on the auxiliary system. If our photon with two DOFs is considered, their Fourier disentangling operations require two controlled operations. However, with our schemes, even if the photon with two DOFs is used as an auxiliary system, we do not need to implement controlled operations or high-dimensional operations on the auxiliary system. Our disentangling operations are only single-qubit operations. Moreover, the Toffoli gate may be realized on different quantum systems, which may be very useful depending on the specific requirements. Different from the Toffoli gate^34^ on the three-atom system, our Toffoli gate may be implemented on a hybrid photon and spin system. Our optical cavity system is easier than the Toffoli gate^35^ using the double-side cavity system. Compared with their six controlled qubit operations^34^,^35^,^63^, our circuits are also compact by as a result of the auxiliary high-dimensional system and cost only three controlled qubit operations. Our theoretical results show that photons and quantum spins may be used alternatively in quantum information processing. Of course, the optical selection rules may be affected by the cavity leakage and spin coherence in quantum dots or the exciton coherence in the experiment. With the recent experiments regarding QD-cavity system^47^,^48^,^49^,^50^,^51^,^52^,^53^,^54^ and the quantum gate between a flying optical photon and a single trapped atom^32^, our results are expected to be applicable for large-scale quantum computation.

## Method

### Optical selection rules

A singly charged GaAs/InAs QD^32^,^33^,^37^,^38^,^39^,^40^,^41^,^42^,^43^,^44^,^45^ has four relevant electronic levels 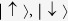, 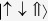, and 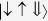. An exciton consisting of two electrons bound to one hole with negative charges can be created by the optical excitation of a photon and an electron spin. In theory, consider the interaction between a single cavity mode and a single two-level spin interacting with a single cavity mode at optical frequencies. By neglecting the spatial dependence^37^,^44^,^45^, taking into account the coupling through the cavity decay channel and neglecting the spatial dependence, the master equation of the whole system can be expressed by the Lindblad form


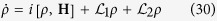


where **H** = **H**_1_ + **H**_2_ + **H**_3_. *ρ* is an arbitrary system operator. 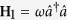 is the Hamiltonian of the input photon pulse. 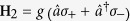 is the standard Jaynes-Cummings Hamiltonian for a two-level system interacting with a single electromagnetic mode by applying the rotating wave approximation and dropping the energy nonconserving terms. 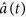 are cavity input operators with the standard commutation relations 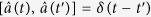. *σ*_−_ and *σ*_+_ are the Pauli raising and lowering operators respectively. 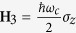 is the system Hamiltonian for the dipole, *ω*_*c*_ is the resonant frequency of the dipole, and *σ*_*z*_ is the Pauli operator for the population inversion. *κ* is the decay rate of the cavity field due to ohmic losses in the metal. 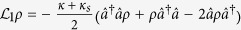 accounts for the damping of the input photon pulse. *κ*_*s*_ is the decay rate of the cavity side leakage mode due to scattering into free-space modes. The scattering rate *κ*_*s*_ may be calculated classically from the Larmor formula. 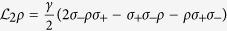 accounts for spontaneous emission of the dipole. Using this Hamiltonian, the Heisenberg equations for first order field/spin moments easily follow


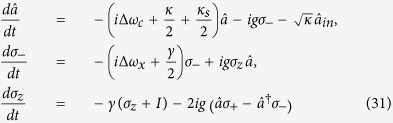


where Δ*ω*_*c*_ = *ω*_*c*_ − *ω* and 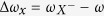, 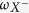 is the frequency dipole transition. The classical boundary condition is defined as 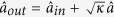
37,38,39 with the input and output field operators 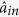 and 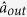, respectively. In the approximation of weak excitation (*X*^−^ stays in the ground state for the most time^39^,^40^,^41^,^42^,^43^), i.e., 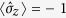, 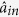 and 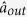 are approximately related with the reflection coefficient


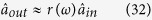


where *r*(*ω*) is defined in equation (27). If the quantum dot is uncoupled from the cavity (*g* = 0), *r*(*ω*) is reduced to *r*_0_(*ω*) as shown in equation (28). For the strong coupling regime *g* ≫ (*κ*, *γ*), one can get |*r*| ≈ 1 and |*r*_0_| ≈ 1 under resonant conditions by adjusting *ω*, *ω*_*x*_ and *ω*_*c*_. Thus, if the excess electron spin lies in the spin state 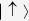, the input light 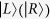 acquires a phase shift of *θ* = arg[*r*(*ω*)](*θ*_0_ = arg[*r*_0_(*ω*)]) by passing through the cavity. Conversely, if the excess electron spin lies in the spin state 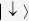, the input light 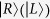 acquires a phase shift of *θ* = arg[*r*(*ω*)](*θ*_0_ = arg[*r*_0_(*ω*)]) by passing through the cavity. Thus, two phase shifts may be obtained as^37^,^38^,^39^


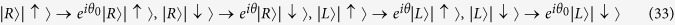


When the side leakage and cavity loss are ignored, the optical selection rules shown in equation (1) are followed by adjusting frequencies to achieve the phase shifts *θ*_0_ = *π* and *θ* = 0^32^,^33^,^44^,^45^.

### Measurement of the entangled excess electron spin in a QD-cavity

To complete our Toffoli gates, the entangled excess electron spins have to be measured under the basis 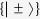. Generally, an auxiliary photon 
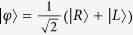
 is used^32^,^33^,^34^,^35^,^44^,^45^. Consider a generally entangled system 
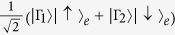
, where 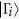 are orthogonal states of other systems except the electron spin *e*. The joint state is first represented by one Hadamard transformation *W*, i.e., 
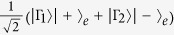
. Then, let the auxiliary photon pass through one circularly polarizing beamsplitter, the QD-cavity, and the other circularly polarizing beamsplitter. This joint system becomes 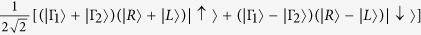. Thus, by measuring the photon under the orthogonal basis 
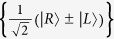
 with one half waveplate, one circularly polarizing beamsplitter and two single photon detectors, the electron spin *e* can be faithfully disentangled. The experimental performances depend on the experimental optical selection rules shown in equation (1).

## Additional Information

**How to cite this article**: Luo, M.-X. *et al.* Hybrid Toffoli gate on photons and quantum spins. *Sci. Rep.* **5**, 16716; doi: 10.1038/srep16716 (2015).

## Figures and Tables

**Table 1:** The relations between the measurement outcomes of the auxiliary photon *D* and the feed-forward operations for implementing the Toffoli gate on three photons *A*, *B* and *C*.

Qubit	Feed-forward	
	Photon *A*	Photon *B*
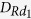	*I*^*p*^	*I*^*p*^
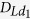	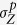	*I*^*p*^
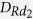	*I*^*p*^	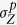
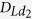	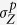	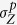

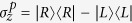
 and 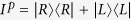.

**Table 2:** The relations between the measurement outcomes of the auxiliary photon *D* and the feed-forward operations for implementing the Toffoli gate on three electron spins *e*_1_, *e*_2_ and *e*_3_.

Qubit	Feed-forward	
	Spin *e*_1_	Spin *e*_2_
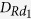	*I*^*e*^	*I*^*e*^
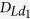	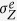	*I*^*e*^
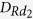	*I*^*e*^	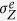
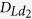	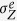	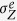

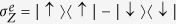
 and 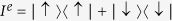.
